# Adapting and translating the Mullen Scales of Early Learning for the South African context

**DOI:** 10.4102/sajcd.v65i1.571

**Published:** 2018-03-08

**Authors:** Juan Bornman, MaryAnn Romski, Kerstin Tonsing, Rose Sevcik, Robyn White, Andrea Barton-Hulsey, Refilwe Morwane

**Affiliations:** 1 Centre for Augmentative and Alternative Communication, University of Pretoria, South Africa; 2 Department of Communication, College of Arts and Sciences, Georgia State University, United States; 3 Department of Psychology, College of Arts and Sciences, Georgia State University, United States; 4 Research in Neurodevelopmental Disabilities Lab, Georgia State University, United States

## Abstract

**Background:**

South African speech-language therapists have identified the need for culturally valid and sensitive assessment tools that can accommodate multiple languages and cover a reasonable age range. The Mullen Scales of Early Learning (MSEL) extend from birth to 68 months, contain five separate subscales including receptive language, expressive language, gross motor, fine motor and visual reception scale, are straightforward to administer and have been successfully used in other African countries, such as Uganda. It also identifies a child’s strengths and weaknesses and provides a solid foundation for intervention planning.

**Objectives:**

This research aimed to demonstrate the appropriateness and usefulness of the translated and culturally and linguistically adapted MSEL across four South African languages (Afrikaans, isiZulu, Setswana and South African English) through two sub-aims: (1) to describe differences, if any, in MSEL performance across language groups and (2) to describe differences, if any, in MSEL performance between age groups.

**Method:**

A total of 198 typically developing children between the ages of 21 and 68 months spread across the four language groups were individually assessed with the culturally and linguistically adapted and translated MSEL.

**Results:**

A one-way analysis of variance (ANOVA) showed no statistically significant differences between the four language groups for total MSEL scores. A Welch’s one-way ANOVA showed that the total MSEL scores were significantly different between age groups.

**Conclusion:**

The translation and adaptation of the MSEL was successful and did not advantage or disadvantage children based on their home language, implying that linguistic equivalence was achieved. The MSEL results differed between age groups, suggesting that the measure was also successful in differentiating the performance of children at different developmental levels.

## Introduction

Finding reliable and valid ways to assess and profile developmental skills in children is an essential step in ultimately developing and providing effective and meaningful interventions. Measuring the effectiveness of intervention, comparing different intervention approaches and/or intensities and being able to predict the effect of additional risk and opportunity factors on children’s development all require reliable and valid assessment tools and methods (Kammerer, Isquith & Lundy, 2003).

Speech-language therapists in South Africa have identified the need to develop culturally valid and sensitive assessment tools for this context (Barrat, Khoza-Shangase & Msimang, 2012; Kathard et al., 2011; Pascoe & Norman, 2011). Attempts to address this need have included the translation, adaptation and renorming of existing test material (Bornman, Sevcik, Romski & Pae, 2010; Gonasillan, Bornman & Harty, 2013; also see Mphahlele, 2006, for a summary) or the development of new assessment materials (Bortz, 1992, 1997; Rangaka, 1999; also see Mphahlele, 2006, for a summary).

The development of new assessment material may, in some ways, be superior to the translation of existing material, as such materials are typically based on locally developed norms and procedures (Pascoe & Norman, 2011). In South Africa, attempts have been made to collect normative data through language samples and observations around certain aspects of child language development within diverse cultural and linguistic groups. For example, Bortz (1992) collected data on the language abilities of 18-month-old isiZulu-speaking children, while Mtshazo (1988) did the same for 3-, 4- and 5-year-old isiZulu speakers. Kvalsig, Liddell, Reddy, Qotyana and Shabalala (1991) studied the communication and teaching situations occurring in the homes of isiZulu- and Sesotho-speaking preschoolers, while Ligthelm (2001) studied the interaction patterns between black South African caregivers and their children. Suzman (1990) collected longitudinal naturalistic language samples from isiZulu-speaking children between the ages of 1 year 10 months and 3 years 5 months and described the children’s grammatical development. Various studies have also been conducted to collect data on the language development of Afrikaans-speaking children (e.g. Vorster, 1983). These have resulted in the Afrikaans version of the language assessment, remediation and screening procedure (LARSP) (Penn & Jordaan, 2016).

Language tests have also been developed and normed for some of the other South African languages (see Mphahlele, 2006, for a summary), with more development in Afrikaans than in any other language. The only language assessment instruments known to the authors that were developed to cover various South African languages are the South African Language Assessments (SALA; Bortz, 1997). Bortz originally devised this test in isiZulu (Zulu Expressive Receptive Language Assessment; Bortz, 1992, based on knowledge of isiZulu grammar and language samples from young children. The test was normed on 303 isiZulu-speaking children aged 3 years 9 months to 4 years 2 months. This test was then translated into Setswana, Sesotho, Tshivenda and Shangaan, although it was not normed for these languages.

While longitudinal large-sample studies to gather developmental data from typically developing children in specific contexts and from specific cultural and language groups would certainly be desirable as a credible foundation for developmental assessments, funding, time, access, human resource and other limitations hardly permit such studies to be executed in low- and middle-income countries (Kammerer et al., 2013). Test translation and adaptation can be an alternative, provided that a rigorous process is followed to adequately address linguistic and cultural factors (Bornman et al., 2010; Peña, 2007). This method, whereby an existing test is translated and then normed on a population from a different language background, has often been used in response to the lack of assessment material in South African languages. For example, the *Peabody Picture Vocabulary Test* (*PPVT*) was translated, adapted and re-normed for Northern Sotho-speaking children (Pakendorf, 1996); the Reynell Language Development Scales III for Afrikaans-speaking toddlers (Oosthuizen, 1999) and portions of the *Mullen Scales for Early Learning* (*MSEL*) (Van Rooyen, 2004; Visser, 2004) and the *Ages and Stages Questionnaire* (Louw, 2004) for Afrikaans-speaking children. However, the complexity involved in test translation in terms of the test equivalence between the different languages is indeed challenging (Haupt & Alant, 2002; Pakendorf & Alant, 1997). Pakendorf (1996) evaluated and published guidelines to be considered in translating and adapting language assessment materials for the South African context. Based on this procedure, various translations and renorming of language tests have produced some normed assessments. However, while a number of different tests have been translated into South African languages, there has not been a test that has been consistently translated across all languages (Mphahlele, 2006).

The MSEL (Mullen, 1995) are individually administered, norm-referenced measures. They have been used to assess the development in young children in other countries in Africa. For example, the MSEL have been employed effectively to evaluate the effects of interventions to improve cognition in Ugandan children exposed to human immunodeficiency virus (HIV) (Boivin et al., 2013; Lorencz & Boivin, 2013). Bodeau-Livinec, Cot, Koura and Boivin (2013) also employed the MSEL to assess the cognitive skills of children in Uganda for a study of maternal anaemia and its effect on child development. These authors concluded that the scales showed promise as a valid assessment measure, covered a reasonable age range (birth to 68 months) and were straightforward to administer. They can be completed by clinicians (speech-language pathologists, occupational therapists and physiotherapists) and teachers and, on average, take 15 min to complete for 12-month-old children, 25–35 min for 36-month-old children and 40–60 min for 60-month-old children (Mullen, 1995). This instrument contains separate scales for receptive and expressive language, as well as three other scales (gross motor, fine motor and visual reception). T-scores (a commonly used standardised test statistic with mean of 50 and a standard deviation of 10) can be obtained for individual scales, and an optional Early Learning Composite serves to provide an overall estimate of cognitive functioning. The MSEL, therefore, allow the examiner to obtain a general developmental profile of a child as well as information on receptive and expressive language skills, gross and fine motor skills, and visual reception skills. No standardised tools have yet been designed to assess infant development in the population of South African children, although attempts have been made to determine whether the Bayley Scales of Infant Development III could be used on black African urban infants in South Africa (Rademeyer & Jacklin, 2013). The focus of this article, however, is on the MSEL as a broad developmental measure and specifically its applicability as a language measure.

In view of the significant need for the development of culturally valid and reliable communication assessment tools that can accommodate multiple languages and that cover a reasonable age range, translation and cultural adaptation of the MSEL (Mullen, 1995) was undertaken for four South African languages. The aim of this study was therefore to translate and adapt the MSEL (Mullen, 1995) across four South African languages, namely, Afrikaans, isiZulu, Setswana and South African English, and pilot test it in a South African context. Two sub- aims were formulated to address this aim: (1) to compare performance across language groups and (2) to compare performance across age groups.

## Method

### Participants

Participants were recruited from 20 early learning centres in low-income urban and semi-urban areas of Gauteng. These centres were selected on the basis of using either Afrikaans, isiZulu, Setswana or English as the language of teaching and learning. In Gauteng, these four languages are among those spoken most frequently as a first language, with 13.3% of the population speaking English, 12.4% speaking Afrikaans, 10.8% speaking isiZulu and 9.9% speaking Setswana. They also rank within the top six most frequently spoken first languages nationally, with isiZulu being spoken by 22.7% of the total population, Afrikaans by 13.5%, English by 9.6% and Setswana by 8% (Statistics South Africa, 2012). Other languages in the top six include isiXhosa (16%) and Sepedi (9.1%). The fact that the four languages targeted in the study represent the first language of only slightly more than 50% of the population reflects the extent of South Africa’s multilingual reality. These early learning centres differed in size, but all provided a basic play-based child development curriculum. Permission was obtained from the Director of African Self Help Trust (ASHA) Pre-School Association in South Africa who provided us with the relevant schools we could conduct the research.

A total of 205 children with typical development (as determined by teacher report) ranging in age from 21 to 68 months (mean = 45.9 months) were recruited for this study. Although the MSEL can be used with children from birth, 21 months was selected as the entry point as many children start attending early learning centres at this age. We recruited children whose home language was the same as the language used for teaching and learning in the early learning centre they attended. This resulted in 57 children in the Afrikaans group, 54 in the isiZulu group, 52 in the Setswana group and 42 in the English group. Attempts were made to increase the sample size of the English-speaking group, but we were unsuccessful in recruiting more children who spoke English at home and who attended an early learning centre in the catchment area of the research. Children also had no known visual or hearing problems and no report of developmental delay in any area as reported by either the parent or the teacher. Of these 205 children, seven children (3%) did not complete the assessment because of a number of reasons, including not being interested or cooperative or requesting to stop, resulting in a total of 198 children (97%) completing the assessment. A 1:1.1 (male:female) ratio was recorded with 24:29 (boys:girls) in the Afrikaans group, 29:23 in the isiZulu group, 26:25 in the Setswana group and 17:25 in the English group, respectively. Table 1 provides the numbers and mean ages in each group. At least five children per age band per language were included.

**TABLE 1:** Participant age groups across languages.

Age group in months	*n*	Afrikaans (*n* = 53)		isiZulu (*n* = 52)		Setswana (*n* = 51)		English (*n* = 42)		Overall (*N* = 198)	
		M	SD	M	SD	M	SD	M	SD	M	SD
21–26	22	24.3	2.6	24.4	0.9	24.6	0.5	23.7	1.6	24.2	1.6
27–32	25	30.8	1.8	30.4	1.1	30.4	2.3	29.2	2.2	30.4	1.7
33–38	23	34.6	1.5	36.3	1.9	35.0	1.2	34.6	1.1	35.3	16
39–44	21	42.4	1.9	41.4	1.9	42.0	0.9	42.2	0.8	42.0	1.4
45–50	25	49.7	0.5	47.6	1.6	47.0	1.5	46.4	1.1	47.9	1.7
51–56	27	54.0	1.4	53.8	1.8	53.3	2.6	54.8	1.1	54.0	1.7
57–62	29	59.4	1.7	59.6	1.7	59.0	1.4	59.2	2.2	59.3	1.6
63–68	26	65.6	1.5	65.1	1.2	65.4	2.3	65.0	2.1	65.3	1.7
Overall	198	47.6	13.8	45.4	13.6	45.1	13.2	45.0	14.1	45.7	13.6

N, number, M, mean; SD, standard deviation; SE, standard error.

### Material

#### Mullen Scales of Early Learning

The MSEL provide an overall measure of cognitive ability for children from birth to 68 months of age through four subscales (fine motor, visual reception, receptive and expressive language) and an optional fifth gross motor scale that only measures skills to 36 months (Mullen, 1995). The inclusion of receptive and expressive language subscales means that a specific indication of children’s language abilities can also be obtained. The first four scales are referred to as the cognitive scales from which an Early Learning Composite score can be derived. These were the only scales that were interpreted in this study. The MSEL were selected for use in this study, as Bornman et al. (2010) had already conducted a pilot study to adapt these scales for the South African context with 47 typically developing children (aged 36 months [*n* = 13], 42 months [*n* = 8]; 48 months [*n* = 8], 52 months [*n* = 8], 60 months [*n* = 10] from Afrikaans-speaking backgrounds (Bornman et al., 2010).

The MSEL are performance-based assessments, and the 124 items are unique to the specific subscale. Each scale is comprised of interactive tasks that can be completed by the child. For example, the gross motor scale includes standing, walking and running; the visual reception scale includes matching, sorting and nesting cups; the fine motor scale includes stacking blocks, drawing and stringing beads; the receptive language scale includes recognising body parts and following commands; and the expressive language scale includes answering questions and completing analogies. The response format depends on the item type, and items had been carefully developed to pose a challenge only in the modality being assessed (e.g. the receptive language scale does not require verbal expression) (Mullen, 1995). The item scoring also varies according to the specific item. The MSEL include many items where 1 indicates a correct response and 0 indicates an incorrect response. Other items include 1, 2, 3, 4 or 5 possible score points, and on some items the task scores must be summed to obtain the item score (Mullen, 1995). The scale items are presented in hierarchical order of difficulty, and scale administration is discontinued after three consecutive wrong responses (ceiling level is reached). A basal level is established if the child scores at least one point on three consecutive items. The maximum achievable raw score per scale is 50 for the visual reception scale, 49 for the fine motor scale, 48 for the receptive language scale and 50 for the expressive language scale. However, it should be noted that younger children are not expected to achieve maximum scores on the scales in order for their skills to be considered age appropriate, as this is a developmental measure.

We translated all five of the MSEL (Mullen, 1995) subscales culturally and linguistically into four languages frequently spoken as the home language in the northern region of South Africa: Afrikaans, isiZulu (from the Nguni language group), Setswana (from the Sotho language group) and South African English (e.g. using ‘nappy’ as opposed to ‘diaper’) using a rigorous translation procedure.

To ensure that we translated the assessment both culturally and linguistically into each language, we employed the guidelines recommended by Peña (2007). We used a process of forward translation, blind-back translation and subsequent review panel to translate all scales with instructions (Bornman et al., 2010; Peña 2007). Two bilingual translators for each language group translated the MSEL from English (source language) into the target language. Two other bilingual translators then translated the target language back into the source language. A six-person review committee (four translators and two of the authors) compared the two English versions. This strategy was repeated for each of the four languages. All of the translators were therapists (speech-language, occupational or physiotherapists) or formally qualified teachers employed at a tertiary institution with at least 10 years’ of experience in working with children. Discrepancies were discussed, resolved and incorporated into the assessment measure by consensus. Equivalence across all four languages was also ensured by comparing the four different source language translations (e.g. as the word ‘purple’ did not exist in either isiZulu or Setswana, it was replaced by the word ‘pink’ in all four languages). The review committee ensured that cultural adaptations (e.g. inches to centimetres and dollars to rands) were incorporated into the assessments in each language. Changes were also made to reflect the use of appropriate objects and materials for the context (e.g. using South African coins). Two teachers and two speech-language therapists served as an expert panel and reviewed the adapted scales to ensure functional equivalence. Minor changes were suggested and incorporated into the final versions of the adaptation.

### Data collection

The MSEL were administered by either formally qualified teachers or therapists (speech-language therapists and occupational therapists) registered, respectively, with the South African Council for Educators (SACE) or Health Professions Council of South Africa (HPCSA), who underwent the Collaborative Institutional Training Initiative (CITI) training on ethical issues relating to human participant research. The 12 different examiners were all employed at a tertiary institution, had a minimum of 7 years’ experience in assessing children and were all trained in the scoring of the MSEL.

Children were assessed individually according to the guidelines in the Item Administration Book of the MSEL (Mullen, 1995). The measure was administered in the child’s home language following its standardised format by an examiner who was a fluent speaker of the target language and who was suitably trained to complete the assessment. During the assessment, the child and the examiner sat at a child-size table in a quiet room at the centre and while one examiner presented the items to the child, the second examiner who was also trained in the MSEL completed the score sheet based on the child’s responses. After the assessment was complete, the two examiners reviewed and clarified the scoring and resolved any potential differences to obtain one consensus score. Scores for each item (and, where appropriate, scores for individual tasks making up an item) and summed raw scores for each scale were transferred onto Excel spreadsheets. In order to check the reliability of the data captured onto the Excel spreadsheets, an independent reviewer checked all entries against the original score sheets. Overall raw score total per scale was also recalculated using the SUM function in Excel and compared with the raw scores summed manually on the score sheets. Any disagreements between the score sheet and Excel spreadsheet were noted. The percentage agreement was calculated by dividing all agreements by the sum of agreements and disagreements. Percentage agreement amounted to 98.2%, indicating that data were captured reliably. All incorrectly captured scores were corrected. The complete assessment of each participant was videotaped to ensure procedural integrity and for further analysis if needed. Administration took between 25 and 60 min, depending on the age of the child.

### Data analysis

To address the first sub-aim, namely to determine if there were significant differences in MSEL performance between language groups, total MSEL raw scores and total raw scores on each subtest of the MSEL were used in separate one-way analysis of variance (ANOVA). When examining total MSEL raw score between language groups, there were no outliers as assessed by boxplots, and data were normally distributed for each language group as assessed by Q-Q plots. There was homogeneity of variances as assessed by Levene’s test of homogeneity of variances (*p* = 0.390). When examining each subtest of the MSEL (visual reception, fine motor, receptive language and expressive language), there were no outliers for the visual reception, fine motor, receptive language and expressive language subscales as assessed by boxplots, and data were normally distributed for each group as assessed by Q-Q plots. There was homogeneity of variances as assessed by Levene’s test of homogeneity of variances for the fine motor, receptive language and expressive language subtests, respectively (*p* = 0.634, *p =* 0.135, *p* = 0.333). There was not homogeneity of variances as assessed by Levene’s test of homogeneity of variances for the visual reception subtest (*p* = 0.041); hence, the one-way ANOVA for the visual reception subtest was corrected using Welch’s *F*. Welch’s *F* adjusts *F* and the residual degrees of freedom to combat problems arising from violations of the homogeneity of variance assumption (Field, 2009).

To address the second sub-aim, namely, to determine if total MSEL raw score performance and performance on each of the four subscales differed significantly between children from each of the eight age groups (21–26; 27–32; 33–38; 39–44; 45–50; 51–56; 57–62 and 63–68 months), total MSEL raw scores and total raw scores on each subtest of the MSEL were used in separate one-way ANOVAs, each corrected using Welch’s *F*. Welch’s *F* was again used to correct for heterogeneity in variances as assessed by Levene’s test for total MSEL score between age groups (*p* = 0.0005), and all subtests of the MSEL (visual reception, *p* = 0.0005; fine motor, *p* = 0.051; receptive language, *p* = 0.003 and expressive language, *p* = 0.001). Boxplots assessed for total *MSEL* score revealed five outliers (two in the 27–32 months age group and one each in the 33–38, 57–62 and 63–68 months age group). There were six outliers found for the visual reception subscale (four in the 27–32 months age group and one each in the 45–50 and 57–62 months age groups), 11 outliers for the fine motor subscale (one each in the 27–32, 33–38 and 45–50 months age groups and two each in the 51–56, and 63–68 months age groups, and four in the 57 – 62 age group), one for the receptive language subscale in the 63–68 months age group, and seven outliers in the expressive language subscale (two each at 27–32 and 63–68 months, and one each at 39–44, 51–56 and 57–62 months). Games-Howell post hoc analyses were used to compare performance between age groups in all ANOVAs and are robust to interpreting differences between groups of unequal variance and sample size.

## Ethical consideration

Ethics approval was obtained from the Institutional Review Board (IRB) at Georgia State University and the Research and Ethics Committee at the Faculty of Humanities at the University of Pretoria, South Africa. Informed consent was obtained from both principals and teachers at the early learning centres. Information letters were sent to parents to inform them of the details of the study and request their consent for their child to participate in the study. Informed consent was obtained from each parent before an individual child participated. In addition, children were asked to provide assent themselves.

## Results

The results are discussed according to the two sub-aims.

### Differences between the four language groups

Table 2 reports mean total MSEL raw scores, standard deviations, confidence intervals and the range of scores for each language group. There were no significant differences in total MSEL scores between the four language groups included in this study [*F* (3, 194) = 1.202, *p* = 0.310].

**TABLE 2:** Mean total Mullen Scales of Early Learning raw scores, standard deviations, confidence intervals and the range of scores for each language group.

Language	*n*	*M*†	SD	SE	95% CI for *M*		Minimum	Maximum
					Lower bound	Upper bound		
Afrikaans	53	146.72	35.36	4.86	136.97	156.46	63.00	188.00
isiZulu	52	136.31	38.11	5.29	125.70	146.92	62.00	188.00
Setswana	51	137.98	35.80	5.01	127.91	148.05	71.00	190.00
English	42	133.12	40.62	6.27	120.46	145.78	57.00	186.00
**Total**	**198**	**138.85**	**37.42**	**2.70**	**133.60**	**144.09**	**57.00**	**190.00**

† , The mean raw score was computed by summing the raw scores obtained for four scales (visual reception, fine motor, receptive language and expressive language) and averaging this sum across participants.

MSEL, Mullen Scales of Early Learning; CI, confidence interval; n, number; M, mean; SD, standard deviation; SE, standard error.

In addition, there were no significant differences in performance between language groups on the four individual MSEL subscales (visual reception, fine motor, receptive language and expressive language). Results of each one-way ANOVA for each MSEL subscale are reported in Table 3.

**TABLE 3:** One-way analysis of variance results for each Mullen Scales of Early Learning subtest by language group.

MSEL subtest	*df*	*F*	*p*
Visual reception	3104.14	1.827†	0.147
Fine motor	3194	0.630	0.596
Receptive language	3194	1.362	0.256
Expressive language	3194	1.732	0.162

† , Welch’s *F*.

MSEL, Mullen Scales of Early Learning.

### Differences between the age groups

Table 4 reports mean total MSEL raw scores, standard deviations, confidence intervals and the range of scores for each age group on the MSEL. Mean MSEL total scores increased in order from low to high between each of the eight age groups. Total MSEL scores were significantly different between age groups [*F* (7, 79.302) = 229.129, *p* < 0.0005]. Post hoc analysis revealed that the mean difference in MSEL total score was statistically significant between each age group except for the difference between the upper age ranges of 51–56 months, 57–62 months and 63–68 months (*p* = 0.857, *p* = 0.109 and *p* = 0.467, respectively).

**TABLE 4:** Mean total Mullen Scales of Early Learning raw scores, standard deviations, confidence intervals and the range of scores for each age group.

Age (in months)	*n*	*M*†	*SD*	*SE*	95*%* CI for *M*		Minimum	Maximum
					Lower bound	Upper bound		
21–26	22	78.95	11.97	2.55	73.65	84.26	57.00	102.00
27–32	25	96.00*	17.58	3.52	88.75	103.26	67.00	135.00
33–38	23	114.35*	10.53	2.19	109.80	118.90	92.00	133.00
39–44	21	130.76*	20.27	4.42	121.53	139.99	91.00	164.00
45–50	25	157.00*	13.62	2.72	151.38	162.62	128.00	177.00
51–56	27	162.82	16.48	3.17	156.30	169.33	119.00	185.00
57–62	29	173.10	9.15	1.70	169.62	176.59	146.00	188.00
63–68	26	178.38	9.96	1.95	174.36	182.41	139.00	190.00
Total	198	138.85	37.42	2.66	133.60	144.09	57.00	190.00

† , The mean raw score was computed by summing the raw scores obtained for four scales (visual reception, fine motor, receptive language, expressive language) and averaging this sum across participants;

* , Mean increase is statistically significant, *p* < 0.05.

n, number; M, mean; CI, confidence interval; SD, standard deviation; SE, standard error.

Tables 5–8 present the mean visual reception, fine motor, receptive language and expressive language raw scores, standard deviations, confidence intervals and the range of scores for each age group, respectively. One-way ANOVAs performed for each MSEL subscale revealed significant differences between age groups and are reported in Table 9.

**TABLE 5:** Visual reception Mullen Scales of Early Learning raw scores, standard deviations, confidence intervals and the range of scores for each age group.

Age (in months)	*n*	*M*	*SD*	*SE*	95*%* CI for *M*		Minimum	Maximum
					Lower bound	Upper bound		
21–26	22	20.14	4.91	1.0	17.96	22.31	11.00	29.00
27–32	25	25.52*	7.52	1.50	22.41	28.62	9.00	41.00
33–38	23	31.04*	4.36	0.91	29.16	32.93	24.00	40.00
39–44	21	35.33	6.92	1.51	32.18	38.48	24.00	49.00
45–50	25	39.88*	3.07	0.61	38.61	41.15	31.00	44.00
51–56	27	42.52	3.66	0.70	41.07	43.97	33.00	49.00
57–62	29	41.93	3.01	0.56	40.79	43.08	33.00	46.00
63–68	26	44.42	3.18	0.62	43.14	45.71	38.00	50.00
Total	198	35.62	9.44	0.67	34.30	36.94	9.00	50.00

* , Mean is significantly different from mean of previous age group, *p* < 0.05.

n, number; M, mean; CI, confidence interval; SD, standard deviation; SE, standard error.

**TABLE 6:** Fine motor Mullen Scales of Early Learning raw scores, standard deviations, confidence intervals and the range of scores for each age group.

Age (in months)	*n*	*M*	*SD*	*SE*	95*%* CI for *M*		Minimum	Maximum
					Lower bound	Upper bound		
21–26	22	21.23	2.11	0.45	20.29	22.16	17.00	24.00
27–32	25	25.57*	3.99	0.80	23.91	27.21	18.00	37.00
33–38	23	28.52*	3.01	0.63	27.22	29.82	23.00	35.00
39–44	21	32.62*	4.67	1.02	30.50	34.75	25.00	43.00
45–50	25	39.60*	4.14	0.83	37.89	41.31	28.00	46.00
51–56	27	40.93	5.43	1.05	38.78	43.08	27.00	48.00
57–62	29	44.14	3.95	0.73	42.63	45.64	31.00	49.00
63–68	26	45.38	4.02	0.79	43.76	47.01	30.00	49.00
Total	198	35.36	9.37	0.67	34.05	36.68	17.00	49.00

* , Mean is significantly different from mean of previous age group, *p* < 0.05.

n, number; M, mean; CI, confidence interval; SD, standard deviation; SE, standard error.

**TABLE 7:** Receptive language Mullen Scales of Early Learning raw scores, standard deviations, confidence intervals and the range of scores for each age group.

Age (in months)	*n*	*M*	*SD*	*SE*	95*%* CI for *M*		Minimum	Maximum
					Lower bound	Upper bound		
21–26	22	19.77	4.49	0.96	17.78	21.76	12.00	27.00
27–32	25	24.64*	5.05	1.01	22.56	26.72	15.00	33.00
33–38	23	28.22*	2.75	0.57	27.03	29.40	23.00	33.00
39–44	21	31.76	5.22	1.14	29.38	34.14	23.00	44.00
45–50	25	37.36*	3.88	0.78	35.76	38.96	28.00	43.00
51–56	27	38.63	4.29	0.83	36.93	40.33	30.00	45.00
57–62	29	42.21*	3.51	0.65	40.87	43.54	33.00	46.00
63–68	26	42.70	2.24	0.44	41.79	43.60	35.00	46.00
Total	198	33.73	8.82	0.63	32.49	34.96	12.00	46.00

* , Mean is significantly different from mean of previous age group, *p* < .05.

n, number; M, mean; CI, confidence interval; SD, standard deviation; SE, standard error.

**TABLE 8:** Expressive language Mullen Scales of Early Learning raw scores, standard deviations, confidence intervals and the range of scores for each age group.

Age (in months)	*n*	*M*	*SD*	*SE*	95% CI for *M*		Minimum	Maximum
					Lower bound	Upper bound		
21–26	22	17.82	2.70	0.58	16.62	19.02	14.00	23.00
27–32	25	20.28	4.83	0.97	18.29	22.28	8.00	33.00
33–38	23	26.57*	4.46	0.93	24.64	28.49	16.00	35.00
39–44	21	31.05	6.67	1.46	28.01	34.09	17.00	44.00
45–50	25	40.16*	6.14	1.23	37.62	42.70	29.00	48.00
51–56	27	40.74	6.82	1.31	38.04	43.44	20.00	50.00
57–62	29	44.83	3.91	0.73	43.34	46.32	34.00	50.00
63–68	26	45.88	3.41	0.67	44.51	47.26	36.00	50.00
Total	198	34.14	11.50	0.82	32.53	35.75	8.00	50.00

* , Mean is significantly different from mean of previous age group, *p* < 0.05.

n, number; M, mean; CI, confidence interval; SD, standard deviation; SE, standard error.

**TABLE 9:** One-way analysis of variance results for each Mullen Scales of Early Learning subtest by age group.

MSEL subtest	*df*	Welch’s *F*	*p*
Visual reception	778.86	83.923	< 0.005
Fine motor	780.40	188.043	< 0.005
Receptive language	779.26	128.917	< 0.005
Expressive language	779.86	220.125	< 0.005

Tables 5–8 highlight results from each post hoc analysis for mean differences found between age groups by each MSEL subscale. Mean differences in MSEL visual reception scores for each age group were significantly different from the mean of the previous age group, with the exception of scores at 39–44 months, 51–56 months, 57–62 months and 63–68 months (*p* = 0.260, *p* = 0.113, *p* = 0.998 and *p* = 0.078, respectively). Mean differences in MSEL fine motor scores for each age group were significantly different from the mean of the previous age group, with the exception of scores at 51–56 months, 57–62 months and 63–68 months (*p* = 0.973, *p* = 0.215 and *p* = 0.940, respectively). Mean differences in MSEL receptive language scores for each age group were significantly different from the mean of the previous age group, with the exception of scores at 39–44 months, 51–56 months and 63–68 months (*p* = 0.139, *p* = 0.949 and *p* = 0.998, respectively). Mean differences in MSEL expressive language scores for each age group were significantly different from the mean of the previous age group, with the exception of scores at 27–32 months, 39–44 months, 51–56 months, 57–62 months and 63–68 months (*p* = 0.381, *p* = 0.193, *p* = 1.000, *p* = 0.144 and *p* = 0.960, respectively).

## Discussion

The results obtained from the translated and adapted MSEL did not differ between language groups. This suggests that the translation and adaptation was successful as it did not advantage or disadvantage any group of children based on home language, implying that linguistic equivalence had indeed been achieved (Peña, 2007).

The total MSEL results differed between age groups, showing that the measure can distinguish the performance of children of different ages, as is expected from a developmental measure. This would also strengthen the confidence in the ability of the translated MSEL to measure changes in a child’s skill profile pre- and post-intervention over and above those that are developmentally expected. The measure is less sensitive in detecting differences between children in the upper age ranges. The expressive language subscale is also variable in its age sensitivity.

Overall, the translated MSEL show promise as a developmental assessment tool that can be used with children aged 21–68 months from four South African language backgrounds. A uniform measure across different language groups is helpful for researchers and clinicians alike, as it can assist in ensuring equitable service delivery and equitable inclusion in research projects aimed at service development across different populations. Formal assessment tools such as the MSEL should never be used in isolation. However, combined with a thorough and culturally sensitive understanding of a child’s medical and developmental history, caregiver concerns and observational methods of assessment (Andersson, 2004), the MSEL can nevertheless assist in guiding clinical decisions-making (including service eligibility and discharge decisions) and monitoring progress. Research projects that concern the measurement of the effectiveness of intervention, the comparison of different intervention approaches and/or intensities and the prediction of the effect of additional risk and opportunity factors on children’s development likewise require reliable and valid assessment tools and methods (Kammerer et al., 2003), and the translated MSEL, in combination with other assessment methods, show promise in this regard.

The translated MSEL can be used as a ‘first draft’, which, through additional processes of expert review, piloting, item review, tests–retest and statistical procedures such as item goodness of fit through logistic regression (Gladstone et al., 2008) can be refined to be a reliable and valid assessment tool with suitable norms for children from various South African language backgrounds.

## Limitations

There were some limitations in this study. Firstly, teachers at early learning centres were requested to identify potential participants with typical development. This could have influenced the recruitment process as formal training of teachers at early learning centres has only recently been addressed in policy with the adoption of the National Integrated Early Childhood Development Policy (South Africa, 2015). Secondly, the sample size per age and language group was small and homogeneous in terms of SES and recruitment area; therefore, results obtained are unsuitable for attempting to obtain formal measures of validity and reliability, and also to obtain norms. For example, while mean scores increased between each of the eight age groups across subtests, there was a smaller mean increase in performance between children at older ages, particularly above 51 months of age. The limited change in raw score performance beyond 51 months of age suggests that there may be less sensitivity of the MSEL when used with children between 51 months and 68 months of age. A larger sample size at each age range would permit specific claims about the sensitivity of the MSEL at older ages to be made. Lastly, no further background information (apart from age, home language and language exposure in school) was obtained about the participants. Therefore, it is not possible to rule out the influence of these additional background factors on the findings.

## Conclusion

In conclusion, the translation and adaptation of the MSEL was successful and did not advantage or disadvantage children based on their home language, indicating that linguistic and cultural equivalence was achieved. The MSEL results differed between age groups, suggesting that the measure was also successful in differentiating the performance of children at different developmental levels. Overall, these findings suggest that the MSEL have potential to be used as part of a language assessment battery with clinical populations including children with neurodevelopmental disorders (Romski et al., 2018).
